# Immune Cell Production Is Targeted by Parasitoid Wasp Virulence in a *Drosophila*–Parasitoid Wasp Interaction

**DOI:** 10.3390/pathogens10010049

**Published:** 2021-01-08

**Authors:** Jordann E. Trainor, Pooja KR, Nathan T. Mortimer

**Affiliations:** School of Biological Sciences, Illinois State University, Normal, IL 61790, USA; jordanntrainor94@gmail.com (J.E.T.); pkadaba@ilstu.edu (P.K.)

**Keywords:** parasitoid wasp, virulence strategy, venom, immune cell, *Drosophila*

## Abstract

The interactions between *Drosophila melanogaster* and the parasitoid wasps that infect *Drosophila* species provide an important model for understanding host–parasite relationships. Following parasitoid infection, *D. melanogaster* larvae mount a response in which immune cells (hemocytes) form a capsule around the wasp egg, which then melanizes, leading to death of the parasitoid. Previous studies have found that host hemocyte load; the number of hemocytes available for the encapsulation response; and the production of lamellocytes, an infection induced hemocyte type, are major determinants of host resistance. Parasitoids have evolved various virulence mechanisms to overcome the immune response of the *D. melanogaster* host, including both active immune suppression by venom proteins and passive immune evasive mechanisms. We identified a previously undescribed parasitoid species, *Asobara* sp. *AsDen*, which utilizes an active virulence mechanism to infect *D. melanogaster* hosts. *Asobara* sp. *AsDen* infection inhibits host hemocyte expression of *msn*, a member of the JNK signaling pathway, which plays a role in lamellocyte production. *Asobara* sp. *AsDen* infection restricts the production of lamellocytes as assayed by hemocyte cell morphology and altered *msn* expression. Our findings suggest that *Asobara* sp. *AsDen* infection alters host signaling to suppress immunity.

## 1. Introduction

Parasitoid wasps that infect *Drosophila* are a valuable model for understanding parasite behavior and have provided important ecological and molecular insights into host–parasite interactions [1,2,3]. In this system, parasitoids infect larval *Drosophila*, and following infection, *Drosophila* mount a cellular encapsulation response to overcome the invader [4]. This encapsulation response is highly conserved among arthropods [5,6,7,8,9], and encapsulation ability is an important determinant of pathogen resistance in insect vectors of human disease [10,11,12]. The encapsulation response in *Drosophila melanogaster* is mediated by hemocytes (immune cells), including circulating macrophage-like cells known as plasmatocytes, as well as lamellocytes, a highly specialized infection-induced immune cell subtype [13]. Plasmatocytes are physiologically activated by parasitoid wasp infection and, following activation, they migrate and adhere to the surface of the parasitoid egg [14,15]. Immune stimulation also triggers the production of lamellocytes [16,17], which adhere to the plasmatocyte cell layer and form a melanized capsule around the egg, killing the developing parasitoid [15,18]. There are multiple routes for lamellocyte production, including the transdifferentiation of plasmatocytes in circulation or within sessile populations, as well as differentiation directly from prohemocyte precursors in the lymph gland, the main hematopoietic organ in *Drosophila* [19,20,21]. 

It has been proposed that the main determinant of *Drosophila* immune resistance to parasitoid infection is host hemocyte load [22]. In this context, hemocyte load refers both to the number and activity of hemocytes found in circulation and the potential for the production of additional hemocytes following infection. Studies have found that an increased number of hemocytes confers resistance to parasitoid infection in *D. melanogaster* and other *Drosophila* species [23,24,25,26,27], and that the production and function of lamellocytes is critical for a successful encapsulation response [18,22,27].

*Drosophila*-infecting parasitoid wasps have evolved multiple mechanisms that allow them to evade or overcome the host immune response, the most prevalent of which is the transfer of venom virulence proteins into the host during infection. Because of the importance of hemocyte number for resistance, many of these parasitoid virulence mechanisms target host hemocytes. This includes venom virulence proteins that act on host hemocytes in a variety of ways including inducing hemocyte lysis [28], promoting death of hemocyte precursor cells [29,30], and inhibition of hemocyte function leading to immunodeficiency [14,18,31,32,33,34]. Many of these venom proteins specifically target lamellocytes [17,18,28,34,35], reinforcing the vital role that this hemocyte subtype plays in the encapsulation response. The outcome of these venom activities is to suppress host hemocyte load either by reducing the number or function of these immune cells.

Along with these active immune suppression mechanisms, parasitoids can also use passive immune evasive mechanisms to escape encapsulation [36,37]. Several passive mechanisms have been proposed including the binding of parasitoid eggs to host tissues as a form of camouflage from the immune response [14,36,38]; an increase in parasitoid egg size following infection [39,40]; and superparasitism, where a single host is multiply infected by conspecific parasitoids and has been suggested to increase parasitoid infection success [40,41,42,43].

In the present study, we describe an uncharacterized parasitoid species of the genus *Asobara* (*Asobara* sp. *AsDen*) to gain further insight into *Drosophila*–parasitoid interactions. We found that following infection, the host immune response is induced, but that host lamellocyte development is impaired, allowing the parasitoid to overcome the host immune defense. *Asobara* sp. *AsDen* is related to several well-studied *Asobara* species including *Asobara tabida* and *Asobara citri*, and thus our characterization of *Asobara* sp. *AsDen* can enable additional comparative studies with these species. The conservation of the encapsulation response in human disease vectors and the use of parasitoid wasps as biological control agents makes understanding parasitoid virulence strategies an important research goal.

## 2. Results

### 2.1. AsDen Is a Strain of an Undescribed Asobara Species

Female braconid wasps were caught in Denver, CO, USA in 2014 and allowed to infect the *OstΔ^EY02442^* encapsulation-deficient *D. melanogaster* strain [18]. These infections resulted in an all-female parthenogenetic strain, which was reared in the lab for three years prior to beginning experimentation. We sequenced the cytochrome oxidase I (COI) gene from this wasp strain and compared the sequence to COI sequences from known braconid species. Our sequence analysis suggests that the strain is a previously undescribed species of the genus *Asobara*. We refer to this wasp species using the name *Asobara* sp. *AsDen* or by the strain name *AsDen* to indicate the genus and location of collection.

**Figure 1:**
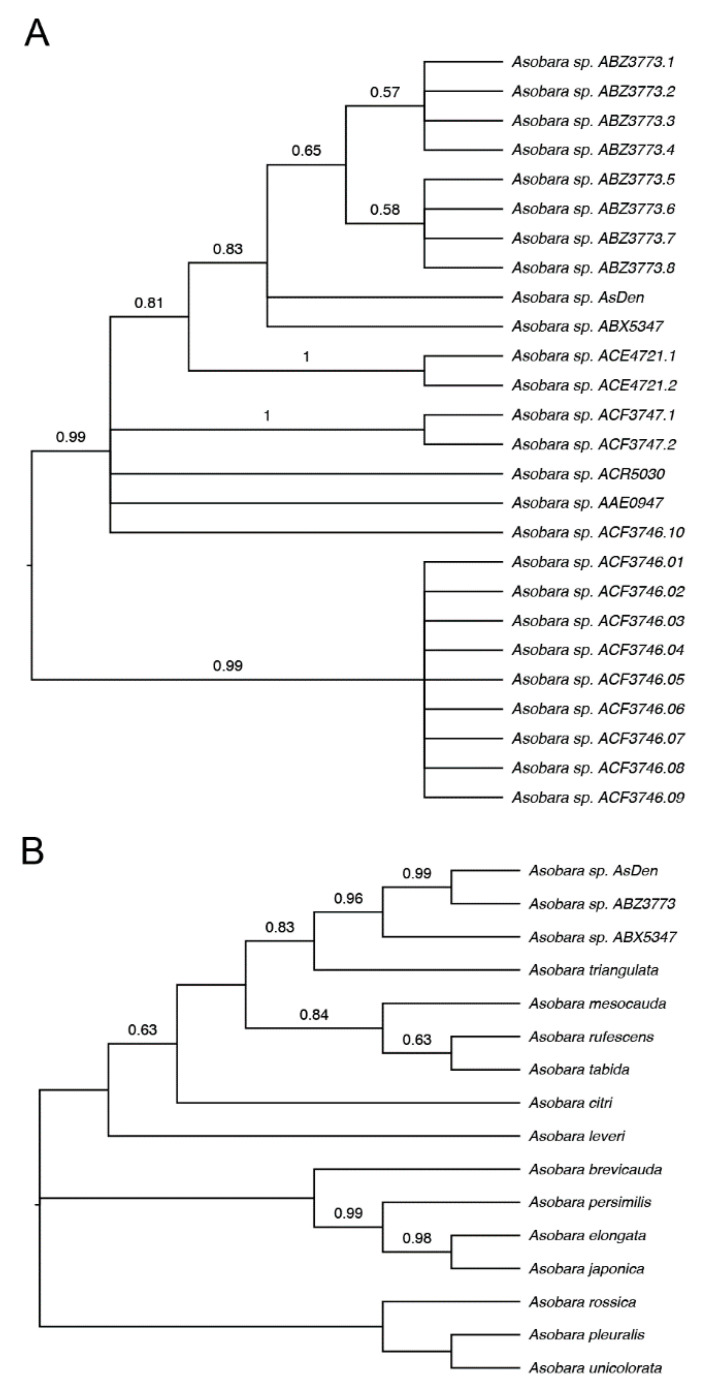
(**A**,**B**) Phylogenetic analysis of the cytochrome oxidase I (COI) gene in *Asobara* sp. *AsDen* with other species of the genus *Asobara*. The evolutionary history was inferred by using the maximum likelihood method as implemented in MEGA, and the tree with the highest log likelihood is shown. The proportion of trees from 1000 bootstrap replicates in which the associated taxa clustered together is displayed, and values below 0.5 are not shown. (**A**) Phylogeny of *Asobara* sp. *AsDen* with sequences from 25 individuals belonging to closely related undescribed *Asobara* species (see Table S1 for sequence information). Strains of the same species have a numerical suffix appended to the species name. (**B**) Phylogeny of *Asobara* sp. *AsDen* with sequences from well-studied species of *Asobara* (see Table S2 for sequence information).

Our basic local alignment search tool (BLAST) analysis of *Asobara* sp. *AsDen* revealed that the most closely related species are additional uncharacterized species of *Asobara* identified in recent efforts to catalog arthropod biodiversity (Table 1) [44,45,46]. In order to further characterize the evolutionary relationships between *Asobara* sp. *AsDen* and these other species, we performed phylogenetic analysis using COI sequences. We found that *Asobara* sp. *AsDen* forms a supported clade with the species *Asobara* sp. *ABZ3773* and *Asobara* sp. *ABX5347* [46] (Figure 1A). Interestingly these species are also found in North America (Table S1), further suggesting a recent evolutionary relationship. Additional phylogenetic analysis with previously studied species of *Asobara* suggests that the species group including *Asobara* sp. *AsDen*, *Asobara* sp. *ABZ3773*, and *Asobara* sp. *ABX5347* is most closely related to *Asobara triangulata*, a species known from a single sample collected in Yunnan, China [47]; *Asobara mesocauda*, a species collected in South Korea and China [47]; and the well-studied species *Asobara rufescens* and *Asobara tabida*, which have both been found in Asia, Europe, and North America [46,47,48] (Figure 1B and Table S2). 

### 2.2. Asobara sp. AsDen Avoided Encapsulation by D. melanogaster Hosts

**Figure 2:**
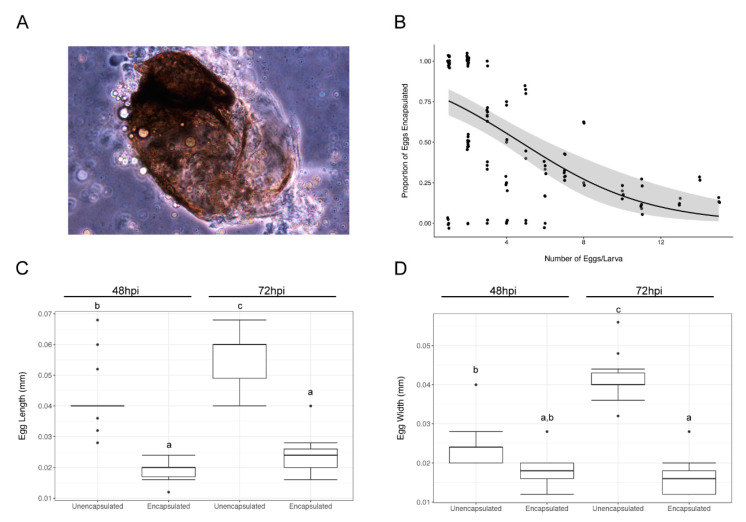
(**A**) Photomicrograph of an encapsulated *AsDen* egg dissected from a *Drosophila melanogaster* host. (**B**) Scatterplot showing the correlation between the number of infections and the proportion of *AsDen* eggs encapsulated in *w^1118^* hosts. Individual data points and the logistic regression line are shown. The 95% confidence interval is shaded in grey and individual data points are jittered on both the *x*- and *y*-axes for clarity. The length (**C**) and width (**D**) of both unencapsulated and melanotically encapsulated *AsDen* eggs were determined at 48 and 72 h post-infection (hpi). Data are displayed as box plots, with calculated outlier data shown as individual points. Letters (a–c) indicate significance groups within each experiment as determined by Tukey’s honest significant difference (HSD).

*AsDen* wasps readily infected *D. melanogaster* larvae, with 98.8% of hosts infected after a 72-h exposure period (*n* = 90 larvae). We found that the *D. melanogaster* immune response successfully encapsulated only 36.6% of *AsDen* eggs (*n* = 372 eggs; an encapsulated egg is shown in Figure 2A). To survive infection, a host must encapsulate every infecting parasitoid egg, and we found that only 34.8% of infected *D. melanogaster* larvae were able to encapsulate all of the infecting *AsDen* eggs (*n* = 89 infected larvae), suggesting a high rate of successful parasitization of *D. melanogaster* hosts by *AsDen*. Interestingly, 77.5% of infected *D. melanogaster* larvae were infected more than once during the exposure period, for an average of 4.2 eggs/infected host larva (*n* = 89 infected larvae). We found a significant negative correlation between the number of eggs laid per larva and the proportion of eggs that are encapsulated (Figure 2B; Pearson’s *r* = −0.58, *p* < 0.001). Taken together, these data suggest that *AsDen* infection triggers the host immune response, but that it is able to successfully overcome host immunity in the majority of infections leading to successful parasitization of *D. melanogaster* hosts. Additionally, multiply infected host larvae are less likely to survive infection.

Similar to other *Asobara* species [39,40], we found that *AsDen* eggs continue to grow in size as they develop in *D. melanogaster* hosts. Eggs were dissected from infected *D. melanogaster* larvae at 48 h post-infection (hpi) and 72 hpi and the length and width of each individual egg was determined. Unencapsulated eggs continue to increase in length (Figure 2C; *t* = 4.30, *p* < 0.001) and width (Figure 2D; *t* = 7.68, *p* < 0.001) between 48 hpi and 72 hpi. To verify that encapsulation was arresting parasitoid development, we determined the length and width of individual encapsulated and melanized eggs at 48 hpi and 72 hpi to compare them with unencapsulated eggs. We found that the melanized eggs were significantly shorter (Figure 2C; *t* = −8.28, *p* < 0.001), and narrower (Figure 2D; *t* = −8.38, *p* < 0.001) than unencapsulated eggs at 72 hpi. Additionally, the increase in size that was seen in unencapsulated eggs was arrested in encapsulated eggs, with no significant size differences observed in encapsulated eggs dissected at 48 hpi and 72 hpi (Figure 2C,D; length: *t* = 1.24, *p* = 0.60; width: *t* = −0.63, *p* = 0.92).

### 2.3. Host lamellocyte Production Is Impaired in Asobara sp. AsDen-Infected Larvae

Many parasitoid species transfer venom virulence proteins to their host during infection to suppress hemocyte number or activity. Often, these virulence proteins target lamellocytes, a parasitoid infection-induced hemocyte subtype that is required for a successful encapsulation response [27,28,29,30]. Lamellocytes are larger and less circular than other hemocytes and can be distinguished from other hemocyte subtypes both by their unique morphology and by the specific expression of *misshapen* (*msn*) [49,50]. Lamellocytes are produced both by the direct differentiation of prohemocytes in the hematopoietic lymph gland and by the transdifferentiation of circulating or sessile plasmatocytes [16,19,20,21], and both routes result in *msn* expression [49].

**Figure 3:**
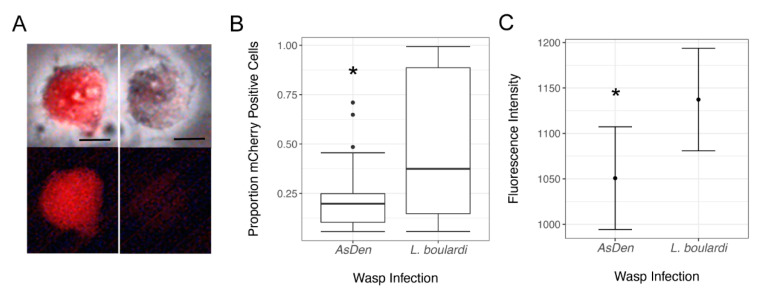
(**A**) Hemocytes dissected from parasitoid-infected *msn-mCherry* larvae. Top panels are merged bright-field and fluorescent images and bottom panels show fluorescence alone. Examples of *msn*-expressing (left) and *msn*-negative (right) hemocytes. Scale bars indicate 10 µm. (**B**) The proportion of hemocytes positive for *mCherry* isolated from *AsDen* and *Leptopilina boulardi*-infected *msn-mCherry* larvae at 72 hpi. Data are displayed as box plots, with calculated outlier data shown as individual points. (**C**) Calculated fluorescence intensity of *mCherry*-positive hemocytes isolated from *AsDen* and *L. boulardi*-infected larvae at 72 hpi. Data are displayed as the mean fit (point) of the effect of parasitoid species on fluorescence intensity ± standard error. In (**B)** and (**C**), * indicates *p* < 0.05 compared to *L. boulardi*-infected larvae.

To assay the production of lamellocytes in *AsDen*-infected larvae, we used a fluorescent cytometer to take high-throughput measurements of cell size, cell perimeter, cell circularity, and *mCherry* fluorescence intensity from hemocytes isolated from infected larvae of the *msn-mCherry* strain (Figure 3A). This strain expresses *mCherry* as a fluorescent reporter of *msn* expression [51]. Because lamellocyte production is induced by parasitoid infection, *msn* is not expressed in the hemocytes of naïve larvae, and thus we used infection with the parasitoid *Leptopilina boulardi* as a comparison for *AsDen* venom activity. *L. boulardi* infection does not inhibit expression of *msn* or lamellocyte development and thus provides a reliable control [17,52,53]. We found that following *L. boulardi* infection, 45.1 ± 4.2% of circulating hemocytes expressed the *msn-mCherry* reporter (*n* = 32,176 hemocytes). In these *L. boulardi*-infected larvae, the *msn-mCherry*-positive cells were larger (cell size: *t* = 29.3, *p* < 0.001; cell perimeter: *t* = 29.4, *p* < 0.001) and less circular (*t* = 21.8, *p* < 0.001) than cells not expressing *msn-mCherry*, consistent with the described properties of lamellocytes [50]. We found that *AsDen* infection also triggered a cellular immune response and lamellocyte production as assayed by *msn-mCherry* expression in *D. melanogaster* hosts. However, only 21.4 ± 1.8% of hemocytes in *AsDen*-infected *msn-mCherry* larvae were *msn*-positive (*n* = 53,908 hemocytes), a significantly lower proportion than observed in stage-matched *L. boulardi*-infected *msn-mCherry* larvae (Figure 3B; z = 7.33, *p* < 0.001). We further found that among the *mCherry*-positive hemocytes, cells from *AsDen*-infected larvae had significantly lower fluorescence intensity compared to cells from *L. boulardi*-infected larvae (Figure 3C; z = 4.84, *p* < 0.001). 

**Figure 4:**
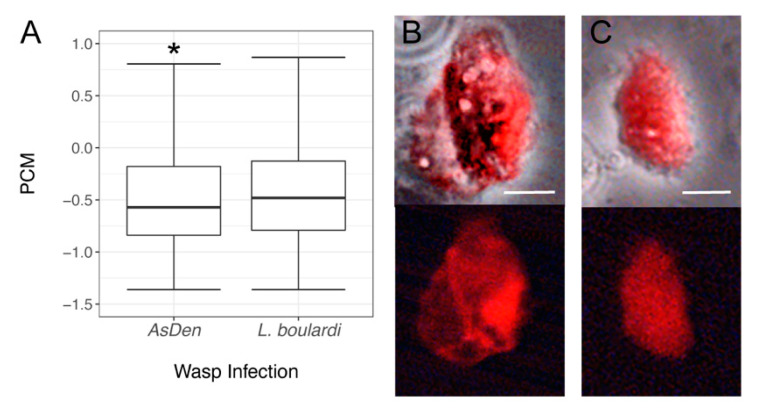
(**A**) Cell morphology principal component analysis (PCM) values calculated from hemocytes isolated from *AsDen* and *L. boulardi*-infected larvae at 72 hpi. Data are displayed as box plots. * indicates *p* < 0.05 compared to *L. boulardi*-infected larvae. (**B**,**C**) Hemocytes dissected from *AsDen*-infected *msn-mCherry* larvae. Top panels are merged bright-field and fluorescent images and bottom panels show fluorescence alone. (**B**) Mature lamellocyte, and (**C**) morphologically abnormal *msn*-expressing immune cell from parasitoid-infected larvae. Scale bars indicate 10 µm.

These differences in *msn* expression may be predicted to result in differences in hemocyte morphology from *L. boulardi* and *AsDen*-infected larvae. To better compare cell morphology between infections, we used principal component analysis (PCA) to reduce the cell size, cell perimeter, and cell circularity measures from the cytometer data to a single dimension. The first principal component of this cell morphology PCA (PCM) had an eigenvalue of 2.35 and explained 78.4% of the variance among these data, suggesting that it accurately captured the data describing hemocyte morphology. We found that PCM values differed significantly between hemocytes from *AsDen* and *L. boulardi* infected larvae (Figure 4A; *t* = 17.03, *p* < 0.001), implying that hemocyte morphology does vary by infection condition. While mature lamellocytes were produced by both *L. boulardi*- and *AsDen*-infected larvae (Figure 4B), most *msn*-expressing immune cells in *AsDen*-infected larvae tended to be smaller and rounder than mature lamellocytes (Figure 4C). This class of *msn*-expressing hemocytes showed an abnormal morphology in comparison with a mature lamellocyte and were not seen following *L. boulardi* infection.

**Figure 5:**
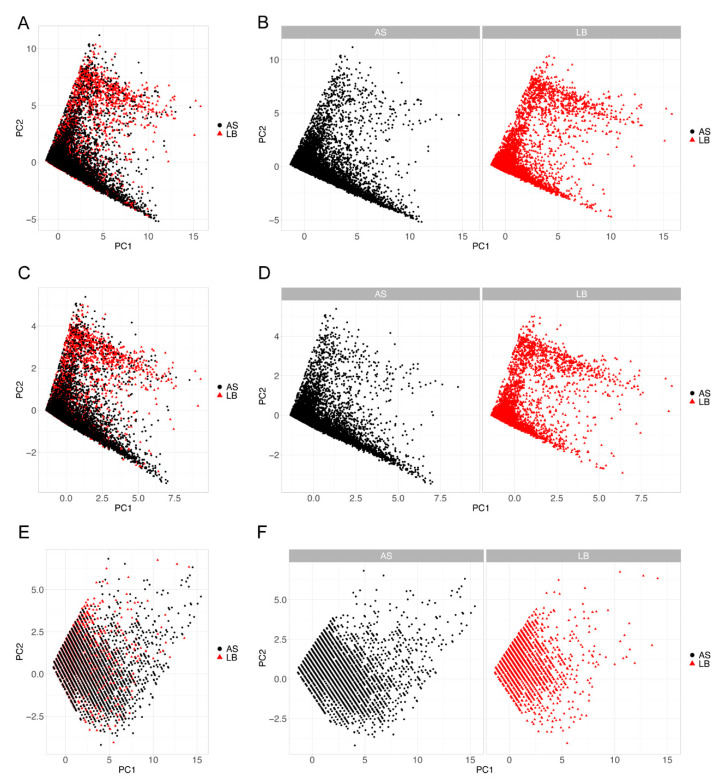
Plots of the first two principal components from a principal component analysis (PCA) of cell morphology and fluorescence intensity performed on (**A**,**B**) all hemocytes, (**C**,**D**) *msn-mCherry*-positive hemocytes, and (**E**,**F**) *msn-mCherry*-negative hemocytes. Hemocytes were extracted at 72 hpi from *msn-mCherry* larvae infected by the indicated parasitoid. Hemocytes from *AsDen*-infected larvae (AS) are shown as black circles and as the left panel of faceted images (**B**,**D**,**F**), and hemocytes from *L. boulardi*-infected larvae (LB) are shown as red triangles and as the right panel of faceted images.

To further characterize the hemocyte populations in *AsDen*- and *L. boulardi*-infected larvae, we performed a second PCA using the previously listed cell morphology features and *mCherry* fluorescence intensity data. We plotted the first two dimensions of this PCA (PC1 and PC2; Table 2), and we found that hemocytes from *L. boulardi*-infected larvae (red triangles in Figure 5A,B) largely clustered into two groups, distinguished by morphology and fluorescence intensity. Although hemocytes from *AsDen*-infected larvae fell into a similar pattern (black circles in Figure 5A,B), one of these groups was greatly reduced. The same pattern was replicated when only data from *mCherry*-positive cells were used for the PCA (Figure 5C,D). However, the PCA plots derived from *mCherry*-negative hemocyte properties were indistinguishable between *AsDen*- and *L. boulardi*-infected larvae (Figure 5E,F). These data supported the hypothesis that *msn*-expressing hemocytes are differentially affected by the parasitoid infections. On the basis of the role of *msn* in lamellocyte production and the observed morphology differences, these data suggest that lamellocyte production is impaired following *AsDen* infection. 

## 3. Discussion

Our findings suggest that a previously uncharacterized parasitoid species from the genus *Asobara*, represented here by the *AsDen* strain, can successfully parasitize *D. melanogaster*. *Asobara* sp. *AsDen* is evolutionarily related to other *Drosophila*-infecting parasitoids including *A. tabida*, although the host ranges of the more closely related, uncharacterized species found in North America are unknown. To characterize the effects of *AsDen* infection on host hemocyte load, and specifically hemocyte morphology and *msn* expression, we compared the properties of hemocytes from *AsDen*-infected hosts to hemocytes from *L. boulardi*-infected hosts. *L. boulardi* infection triggers *msn* expression and lamellocyte production, and *L. boulardi* venom has no known impact on these processes [17,38,52,53], suggesting that this infection can serve as a useful control for our analyses. 

We found that *AsDen* infection triggers an immune response in hosts, but that it has a distinct effect on both hemocyte morphology and *msn* expression in host hemocytes when compared with *L. boulardi* infection. We interpret our findings to suggest that this effect of *AsDen* infection is a result of parasitoid venom activity, as has been shown in similar instances of *Drosophila*-infecting parasitoid wasp virulence [14,28,29,33,34,35]. However, it is possible that this effect is due to an unrecognized parasitoid virulence mechanism, and thus direct experimentation with *AsDen* venom will be necessary to strengthen this conclusion and explore the mechanism in future work.

In the encapsulation response, *msn* is expressed in lamellocytes following infection, and *msn* signaling activity is required for lamellocyte production [51,54]. The proportion of *msn*-positive immune cells is lowered following *AsDen* infection, and *msn* expression levels are decreased in immune cells isolated from *AsDen*-infected larvae in comparison with *L. boulardi*-infected larvae (Figure 3). These findings suggest that *AsDen* infection inhibits host immune signaling, leading to the failure to properly promote lamellocyte specification or development. In agreement with this hypothesis, we observed that many *msn*-expressing cells from *AsDen*-infected larvae display an abnormal lamellocyte-like morphology (Figure 4B,C). Additionally, we found that while hemocytes from *L. boulardi*-infected hosts clustered into two populations on the basis of their morphology and *msn* expression levels, one of these populations was greatly reduced in *AsDen*-infected hosts (Figure 5A,B). Lamellocytes tend to be larger and more irregularly shaped than plasmatocytes [50]. An examination of the factor loading from our cell morphology and fluorescence intensity PCA results (Table 2) suggests that the reduced cell population in *AsDen*-infected larvae tended to be larger, less circular, and *msn*-positive (Figure 5), all of which are consistent with a specific deficit in lamellocyte production. The finding that this alteration in hemocyte characteristics was observed in *msn*-positive cells (Figure 5C,D) but not *msn*-negative cells (Figure 5E,F) further suggests that *AsDen* infection specifically targets *msn* and/or lamellocyte production.

The Msn protein functions in the JNK signal transduction pathway [55]. *msn* is transcriptionally regulated by JNK activity through a positive feedback loop, and thus the *msn-mCherry* reporter strain provides a readout of JNK pathway activity [51]. This suggests that the JNK signaling pathway may be inhibited in *AsDen*-infected larvae. We have yet to determine the molecular mechanism underlying JNK inhibition in *AsDen* infected larvae, but we propose it could act either directly through inhibiting one or more components of the JNK pathway or indirectly by blocking upstream pathway activation to inhibit lamellocyte production. The JNK pathway plays a conserved role in immunity in *Drosophila* and a wide range of species [9,56,57]. In *D. melanogaster*, genes in the JNK pathway are associated with resistance to parasitoids [58,59], and are required for lamellocyte production in response to infection [51]. To our knowledge, *Drosophila* parasitoids have not previously been suggested to inhibit JNK signaling; however, the JNK pathway is targeted by a wide range of other pathogens in a variety of hosts [60,61,62]. 

It is notable that *AsDen*-infected larvae do still produce *msn*-positive hemocytes, suggesting that lamellocyte differentiation and JNK signaling are not completely abolished. Additionally, even though the morphological changes leading to lamellocyte production are impaired in *AsDen*-infected larvae, the cell morphology of *msn*-expressing hemocytes is different from non-*msn*-expressing hemocytes. These data suggest that *AsDen* infection may be inhibiting a specific aspect of lamellocyte transdifferentiation or maturation, consistent with the finding that *msn* expression coincides with early morphological changes in transdifferentiating hemocytes [49]. Recent studies have uncovered a broader range of *Drosophila* hemocyte subtypes than previously appreciated [63,64,65,66], and future investigation into this complexity may help to unravel the specific effects of *AsDen* venom on host hemocytes and lamellocyte production.

These findings suggest that *AsDen* is exhibiting an active immune suppression virulence mechanism. In this proposed mechanism, lamellocyte production is triggered following infection but is suppressed by venom or another parasitoid factor, resulting in the morphologically abnormal lamellocyte-like hemocytes we observed. To our knowledge, these lamellocyte-like cells are not induced during the immune response to other parasitoids, suggesting that they result from the inhibition of lamellocyte development rather than as a typical component of the immune response. In contrast, we would predict that a passive evasion strategy would either fail to trigger any host immune response (including *msn* expression) or result in a reduced number of morphologically normal lamellocytes, rather than the abnormal cells seen following *AsDen* infection.

Along with restricted lamellocyte production or development, *AsDen*- infected hosts have a limited encapsulation response. Interestingly, we found a negative correlation between the number of times a host larva was infected and its encapsulation ability (Figure 2A). Multiple infections of a single host by conspecific parasitoids is known as superparasitism [67], and is commonly observed across many parasitoid species both in laboratory conditions and in nature. The negative effect of superparasitism on host resistance observed in our study may have been due to the additive effects of multiple envenomations on host lamellocyte production; perhaps additional “doses” of venom are able to more completely suppress lamellocyte production. However, we cannot rule out the possibility that superparasitism is acting through an alternative mechanism such as passive immune evasion [36,37]. Supernumerary infections by the parasitoids *Pseudapanteles dignus* and *A. tabida* have been shown to increase the likelihood of successful parasitization [40,42], suggesting that superparasitism itself may contribute to the ability of the parasitoid egg to escape from encapsulation. Parasitoids generally avoid superparasitism and most parasitoid species are able to perceive the presence of eggs from a conspecific female [39,41,68]. In our previous work, we found that using the identical experimental setup with other parasitoid species consistently yields average infection rates of 1–1.2 eggs per infected larva [14,38]. This is in contrast to the 4.2 eggs per infected larva observed for *AsDen* in this study. Many known instances of superparasitism are driven by external factors such viral infections [69,70,71], but this has not yet been determined in this case.

In *Asobara* sp. *AsDen* and many other parasitoid species, virulence appears to be largely driven by a single strategy, for example, the passive immune avoidance of *A. tabida* or the immune suppressive venoms of *AsDen*, *Asobara citri*, *Asobara japonica*, or various species of Figitid parasitoid wasps [30,32,36,72,73,74]. However, both *L. boulardi* and *Ganaspis hookeri* appear to use a combined strategy of venom-mediated immune suppression and passive avoidance [14,37,38], suggesting that further study may uncover more complex virulence strategies across a range of parasitoids than previously appreciated. Further, while *A. tabida* is the most closely related of the well-studied parasitoid species to *Asobara* sp. *AsDen*, its venom has been shown to cause paralysis and inhibit host development with only limited immune-suppressive effects [36,75,76,77,78,79]. This is not entirely unexpected, as other closely related parasitoid species have distinct virulence strategies and venom composition [37,38,80]. It has also been demonstrated that different strains of a single parasitoid species can possess different virulence activities [81,82,83]. As *AsDen* is the only known strain of its species, we were unable to determine how conserved this activity may be with other strains, although this will hopefully be investigated as more strains of this species are identified.

Our findings support the idea that overcoming host hemocyte load is a critical determinant of parasitization success for parasitoid wasps of *Drosophila*. Since *Drosophila* are a valuable model for understanding the immune defenses of insect vectors of human disease and agricultural pests, these findings may provide insight into the interactions between insect vectors and invading pathogens and may have implications for the selection and use of parasitoid wasps in biological control applications.

## 4. Materials and Methods

### 4.1. Insect Strains

Two females from an unknown braconid parasitoid wasp species were collected from a fruit trap in Denver, Colorado, USA, in 2014 and were maintained on the encapsulation deficient *D. melanogaster* mutant strain *OstΔ^EY02442^* (BDSC: 15565) [18] from the Bloomington Drosophila Stock Center. A sub-strain was established from a single parthenogenetic foundress and will be referred to as *AsDen*. The study also uses the parasitoid wasp *Leptopilina boulardi* (strain Lb17) [38], which is maintained in the laboratory on the *Canton S D. melanogaster* strain. The following additional *D. melanogaster* strains were used in this study: *w^1118^* (BDSC: 5905) from the Bloomington Drosophila Stock Center, and *msn-mCherry* [51], provided by Dr. Robert Schulz.

### 4.2. Parasitoid Species Determination

Genomic DNA was extracted from *AsDen* using standard methods. The COI gene was amplified using the “Folmer” primers [84] LCO1490 (primer sequence: GGTCAACAAATCATAAAGATATTGG) and HCO2198 (primer sequence: TAAACTTCAGGGTGACCAAAAAATCA), and sequenced at the UIUC Core Sequencing Facility (Urbana, IL). The resulting Sanger sequencing reads were aligned using 4Peaks software (A. Griekspoor and Tom Groothuis, nucleobytes.com). The *Asobara* sp. *AsDen* COI DNA sequence was submitted to GenBank (accession # MT498809). The resulting DNA sequence was compared against all hymenopteran sequences using the basic local alignment search tool (BLAST) available through the National Center for Biotechnology Information (NCBI) [85]. For further sequence analysis, we constructed a custom BLAST database of all 353 *Asobara* COI sequences available from the NCBI (accessed April 11, 2020) using BLAST+ (version 2.5.0) [86]. This custom BLAST database is available upon request.

### 4.3. Phylogenetics

Phylogenetic analyses were conducted in MEGA X [87,88] using COI DNA sequences. For the first analysis, *AsDen* was compared to the 25 most highly homologous *Asobara* sequences as determined by BLAST+ (Supplemental Table S1) [44,45,46]. For the second analysis, the species group including *AsDen* found in the first analysis was compared against 13 well-studied species of *Asobara* (Supplemental Table S2) [26,47,89,90]. For both analyses, the evolutionary history was inferred by using the maximum likelihood method and Kimura 2-parameter model with 1000 bootstrap replicates [91]. The initial tree for the heuristic search was obtained automatically by applying Neighbor-Join and BioNJ algorithms to a matrix of pairwise distances estimated using the maximum composite likelihood (MCL) approach in MEGA X, and then selecting the topology with superior log likelihood value. Branches corresponding to partitions reproduced in less than 50% of the bootstrap replicates were collapsed. All positions containing gaps and missing data were eliminated. The resulting phylogenetic trees were visualized using FigTree (version 1.4.3, http://tree.bio.ed.ac.uk/).

### 4.4. Parasitoid Infection

For infection with parasitoid wasps, 30 late second instar larvae from the *w^1118^* strain were placed on 35 mm Petri dishes filled with *Drosophila* medium together with 3 *AsDen* wasps at 25 °C. Larvae were dissected at 48 or 72 h post-infection (hpi), as noted. The infected larvae were then scored for the total number of parasitoid eggs and the numbers of encapsulated and non-encapsulated eggs. For size experiments, the length and width of each egg was determined using an E-series Reticle (Leica Microsystems). Egg length was measured from pole to pole and egg width was measured across the widest region perpendicular to the length axis. All experiments were performed in triplicate. 

### 4.5. Expression of msn and Cell Morphology Analyses

The *msn-mCherry D. melanogaster* strain was used to assay expression of *msn*. This strain carries a transgenic construct containing the *msn-F9* enhancer upstream of the *mCherry* red fluorescent protein [51]. Second instar *msn-mCherry* larvae were exposed to either *AsDen* or *L. boulardi* for a 72-h period as described above, with 3 biological replicates for each infection condition. Host hemocytes were isolated 72 hpi and added to a Tali Cellular Analysis Slide (Invitrogen). Hemocytes were allowed to adhere for 30 min and then cell number, size, perimeter, circularity, and red fluorescence intensity were measured using a Tali Image-Based Cytometer (Invitrogen). For each replicate, we imaged 20 fields of cells, with an average of 717.4 cells per field, and a range of 194 to 1455 cells for a total of 32,176 hemocytes from *L. boulardi*-infected larvae and 53,908 hemocytes from *AsDen*-infected larvae. Cytometer data were filtered to only include single cells using the Tali software count function and size-gating, prior to further analysis.

### 4.6. Data Analysis

All statistical analyses were performed in the R statistical computing environment [92] using the multcomp [93], lme4 [94], lmerTest [95], plyr [96], FactoMineR [97], factoextra [98], and ggplot2 [99] packages. Analysis of variance (ANOVA) was used to test the relationship between egg size and time or encapsulation status. Tukey’s honest significant difference (HSD) test was used for multiple comparisons of egg size. Pearson’s product-moment correlation was used to test for correlations between egg number and encapsulation status. Mixed linear models, with replicate as a random effect, were used to test for differences in *msn*-*mCherry* fluorescence intensity and proportion of *mCherry*-positive cells between *AsDen* and *L. boulardi* infections. Welch two-sample *t*-tests were used to compare immune cell morphology data between *AsDen* and *L. boulardi* infections.

To characterize hemocyte populations, we used PCA on the red fluorescence intensity, cell size, cell perimeter, and cell circularity measures from the cytometer data. A circularity value of 1.0 is considered perfectly circular, and values either greater or less than 1.0 are increasingly less circular. To account for this, circularity values were log_2_ transformed and the absolute value of these transformed values were used for PCA. Other measures were used for PCA without transformation. This analysis was repeated separately on gated fluorescence data, generating distinct PCA scores for *mCherry*-positive hemocytes and *mCherry*-negative hemocytes.

## Figures and Tables

**Table 1:** Basic local alignment search tool (BLAST) results comparing the *AsDen* COI DNA sequence against a custom database of 353 *Asobara* COI sequences. The species name, sequence accession number, score (bits), and identity (%) for the top scoring hits by species are displayed.

Species Designation	Accession Number	Score (Bits)	Identity (%)
*Asobara* sp. *ABZ3773*	KR886087.1	974	94
*Asobara* sp. *ABX5347*	JN293161.1	924	93
*Asobara* sp. *ACF3746*	HQ929638.1	913	92
*Asobara* sp. *ACE4721*	JN293665.1	907	92
*Asobara* sp. *ACR5030*	MF936732.1	902	92
*Asobara* sp. *ACF3747*	HQ930298.1	896	92
*Asobara* sp. *AAE0947*	HQ106668.1	891	92

**Table 2:** Eigenvalues and factor loading for the first two dimensions (PC1 and PC2) from PCA of cell morphology and fluorescence intensity of all hemocytes extracted from *L. boulardi*- and *AsDen*-infected *msn-mCherry* larvae, as shown in Figure 4A,B.

Variable	PC1	PC2
Eigenvalue	1.624	0.867
Variance (%)	54.13	28.90
*Factor loading*		
Cell size	0.647	−0.262
Cell circularity	0.636	−0.332
Fluorescence intensity	0.420	0.906

## Data Availability

The data presented in this study are available on request from the corresponding author.

## Supplementary Materials

The following are available online at https://www.mdpi.com/2076-0817/10/1/49/s1: Table S1: Species names, accession numbers and collection location are given for samples used to build the phylogeny shown in Figure 1A. Multiple individuals of a species are listed as independent samples with accession numbers and a numerical suffix appended to the species name. Abbreviations: NP (National Park), SP (State Park). Table S2: Species and strain names, accession numbers and collection location are given for samples used to build the phylogeny shown in Figure 1B.
