# Supplementary material for: Immune Cell Production Is Targeted by Parasitoid Wasp Virulence in a Drosophila–Parasitoid Wasp Interaction
Source: Pathogens. 2021 Jan 8;10(1):49. doi: 10.3390/pathogens10010049 (PMC7826891; doi:10.3390/pathogens10010049)
Supplement: Supplementary file 1 [file pathogens-10-00049-s001.pdf]

pathogens

Table S1. Species names, accession numbers and collection location are given for samples used to build the phylogeny shown in Figure 1A. Multiple individuals of a species are listed as independent samples with accession numbers and a numerical suffix appended to the species name. Abbreviations: NP (National Park), SP (State Park).

Sample Name	Accession #	Collection Location
Asobara sp. ABZ3773.1	KR896844.1	Prince Albert NP, SK, CAN
Asobara sp. ABZ3773.2	KR898757.1	Elk Island NP, AB, CAN
Asobara sp. ABZ3773.3	KR886087.1	Elk Island NP, AB, CAN
Asobara sp. ABZ3773.4	KR875914.1	Prince Albert NP, SK, CAN
Asobara sp. ABZ3773.5	KR884238.1	Wellington County, ON, CAN
Asobara sp. ABZ3773.6	KR888074.1	Elk Island NP, AB, CAN
Asobara sp. ABZ3773.7	KR879657.1	Wellington County, ON, CAN
Asobara sp. ABZ3773.8	KR784633.1	Leeds and Grenville, ON, CAN
Asobara sp. ABX5347	JN293161.1	Yoho NP, BC, CAN
Asobara sp. ACE4721.1	JN293665.1	Glacier NP, BC, CAN
Asobara sp. ACE4721.2	JN292450.1	Glacier NP, BC, CAN
Asobara sp. ACR5030	MF936732.1	Gulf Islands NP, BC, CAN
Asobara sp. AAE0947	HQ106668.1	Restigouche, NB, CAN
Asobara sp. ACF3747.1	HQ930298.1	Bigelow, AR, USA
Asobara sp. ACF3747.2	KR896531.1	Wellington County, ON, CAN
Asobara sp. ACF3746.01	HQ929638.1	Deadhorse Ranch SP, AZ, USA
Asobara sp. ACF3746.02	KY843128.1	Islamabad, Pakistan
Asobara sp. ACF3746.03	KY845150.1	Islamabad, Pakistan
Asobara sp. ACF3746.04	KY838943.1	Islamabad, Pakistan
Asobara sp. ACF3746.05	KY830675.1	Islamabad, Pakistan
Asobara sp. ACF3746.06	KY843285.1	Islamabad, Pakistan
Asobara sp. ACF3746.07	KY832326.1	Islamabad, Pakistan
Asobara sp. ACF3746.08	KY842379.1	Islamabad, Pakistan
Asobara sp. ACF3746.09	KY830249.1	Islamabad, Pakistan
Asobara sp. ACF3746.10	KY840132.1	Islamabad, Pakistan
Asobara sp. AsDen	MT498809.1	Denver, CO, USA

Table S2. Species and strain names, accession numbers and collection location are given for samples used to build the phylogeny shown in Figure 1B.

Species Name	Strain Name	Accession #	Collection Location
Asobara sp. ABZ3773	*ABZ3773*	KR886087.1	Elk Island NP, AB, CAN
Asobara sp. ABX5347	*ABX5347*	JN293161.1	Yoho NP, BC, CAN
Asobara triangulata	*DSZ062*	KT835413.1	Yunnan, China
Asobara mesocauda	*DSZ061*	KT835414.1	Yunnan, China
Asobara rufescens	*TK(1)*	AB920758.1	Tokyo, Japan
Asobara tabida	*AtFr*	JQ808428.1	Sospel, France
Asobara citri	*AcIC*	JQ808423.1	Lamto, Cote d'Ivoire
Asobara leveri	*DSZ084*	KT835427.1	South Korea
Asobara brevicauda	*DSZ066*	KT835453.1	South Korea
Asobara persimilis	*AperAus*	JQ808425.1	Sydney, Australia
Asobara elongata	*DSZ048*	KT835452.1	Yunnan, China
Asobara japonica	*AjJap*	JQ808424.1	Tokyo, Japan
Asobara rossica	*A_rossica*	AB456708.1	Hokkaido, Japan
Asobara pleuralis	*ApIndo*	JQ808427.1	Manado, Indonesia
Asobara unicolorata	*DSZ055*	KT835410.1	Yunnan, China
Asobara sp. AsDen	*AsDen*	MT498809.1	Denver, CO, USA
